# The effect of acute prolonged swimming on skeletal muscle metabolism in different muscle types of rats after nine days of dexamethasone treatment

**DOI:** 10.3389/fphys.2026.1890376

**Published:** 2026-09-22

**Authors:** Damian Jozef Flis, Bartosz Cedro, Alicja Piekarska, Andzelika Borkowska, Malgorzata Halon-Golabek, Ewa Rodziewicz-Flis, Jedrzej Antosiewicz, Wieslaw Ziolkowski

**Affiliations:** 1 Department of Pharmaceutical Pathophysiology, Faculty of Pharmacy, Medical University of Gdansk, Gdansk, Poland; 2 Department of Bioenergetics and Physiology of Exercise, Faculty of Health Sciences, Medical University of Gdansk, Gdansk, Poland; 3 Department of Physiotherapy, Faculty of Health Sciences, Medical University of Gdansk, Gdansk, Poland; 4 Department of Physiotherapy, Gdansk University of Physical Education and Sport, Gdansk, Poland; 5 Department of Rehabilitation Medicine, Faculty of Health Sciences, Medical University of Gdansk, Gdansk, Poland

**Keywords:** dexamethasone, iron metabolism, muscle protein synthesis, oxidative stress, skeletal muscle mass, skeletal muscle proteolysis

## Abstract

**Objectives:**

The study investigated the effec of dexamethasone (Dex) treatment, acute prolonged swimming, and their combination on skeletal muscle mass, oxidative stress, iron metabolism, and AKT-mediated signaling in two muscle types.

**Methods:**

Rats were divided into four groups (n=8 per group): intraperitoneal saline or Dex (1mg/kg/day) treatment for 9 days (non-exercised and exercised). Animals in exercise groups underwent a 4-day preparatory swimming procedure followed by a single 3h swimming session. The soleus (SOL) and extensor digitorum longus (EDL) muscles were collected, weighed, and used for analysis. We assessed lipid and protein peroxidation markers, catalase activity, iron-metabolism proteins, and AKT-dependent proteolysis and synthesis pathways.

**Results:**

Nine days of Dex treatment significantly reduced SOL (-12%, p<0.01) and EDL (-15%, p<0.001) mass, accompanied by oxidative stress (increased protein peroxidation, p<0.001) and alterations in iron metabolism: increased FH (SOL: +88%, p<0.01, EDL: +80%, p<0.01) and decreased PCBP1 (SOL: -59%, p<0.001, EDL: -33%, p<0.05). Dex treatment also affected proteins involved in synthesis and proteolysis pathways: in SOL, lowered total AKT (-22%, p<0.05) and pmTOR (-24%, p<0.05); in EDL, decreased MuRF-1 (-30%, p<0.01) and total mTOR (-24%, p<0.01), highlighting mechanisms underlying muscle loss. In saline-treated rats, acute prolonged swimming, following 4 days of preparatory exercise, did not affect SOL and EDL mass or oxidative stress markers. However, in SOL, swimming reduced PCBP1 (-43%, p<0.001), decreased total AKT (-33%, p<0.01) and pAKT (-47%, p<0.01), increased Atrogin-1 levels (+38%, p<0.001), and decreased pmTOR (-52%, p<0.001) and p70S6K levels (-47%, p<0.05). In EDL, swimming lowered MuRF-1 levels (-23%, p<0.01), indicating specific responses to exercise across muscle types. Dex-induced muscle mass loss was lower after a 4-day preparatory exercise procedure followed by prolonged exercise (p<0.001 for both muscles). Swimming in Dex-treated rats decreased pAKT (-52%, p<0.01) in SOL and increased pmTOR (+52%, p<0.01) in EDL.

**Conclusion:**

Dex treatment reduces skeletal muscle mass, regardless of fiber composition. These changes relate to oxidative stress and an imbalance in iron metabolism, but differences between muscle types in the proteolysis/synthesis signaling pathway highlight contrasting responses across muscle types. Acute prolonged swimming, preceded by a 4-day preparatory procedure, attenuates the adverse effects of Dex treatment.

## 1 Introduction

Glucocorticoids (GCs), particularly dexamethasone (Dex), have been increasingly used since the 1950s, with rising treatment prevalence in adults, notably during the COVID-19 pandemic, to reduce mortality in SARS-CoV-2 infections (Benard-Laribiere et al., 2017; Waljee et al., 2017; Ferreto et al., 2021). Although the beneficial effects of GC treatment are now well known and accepted, controversies remain about their prescription because of conflicting safety results (Bordag et al., 2015). Previous studies have reported that Dex treatment may reduce body weight, decrease muscle strength and mass, reduce protein synthesis, and increase blood lipid levels (De Vos et al., 1995; Flis et al., 2022). Interestingly, Dex induces atrophy of both fast-twitch and slow-twitch fibers (Ahtikoski et al., 2004; Jhuo et al., 2023), mediated by activation of the ubiquitin-proteasome system and disruption of intracellular iron homeostasis, leading to intracellular iron accumulation (Jeong et al., 2025). Excess iron accumulation in muscle cells stimulates reactive oxygen species production and triggers iron-dependent oxidative stress (Ikeda et al., 2016), suggesting another route by which iron imbalance can lead to skeletal muscle atrophy. Dex also increases excessive activity of the ubiquitin-proteasome system regulated by Forkhead box O (FoxO) proteins, which is accompanied by the breakdown of muscle proteins and inhibition of protein synthesis via the Mammalian Target of Rapamycin/Ribosomal protein S6 kinase (mTOR/p70S6k) pathway (Chang and Kong, 2020; Jhuo et al., 2023), and the above-mentioned processes are related to Protein Kinase B (AKT) signaling. In our study, we focused on the quantitative analysis of proteins involved in iron metabolism in muscle cells, including those responsible for iron storage (ferritin L (FL) and ferritin H (FH)), intracellular trafficking (Poly(rC)-Binding Protein 1 (PCBP1) and Poly(rC)-Binding Protein 2 (PCBP2)), and iron export (ferroportin (FPN1) and Amyloid Precursor Protein (APP)). Ferritin, composed of heavy (FH) and light (FL) chains, stores iron in a non-toxic form and exhibits antioxidant properties. The heavy chain (FH) possesses ferroxidase activity, catalyzing the oxidation of Fe^2+^ to Fe^3+^, enabling its safe mineralization and storage within the ferritin shell by the light chain (FL) (Arosio and Levi, 2002; Chen et al., 2020). PCBP1 and PCBP2 were initially identified as RNA-binding molecules but are now recognized as cytosolic iron chaperones. They bind Fe^2+^ and mediate its selective transfer between transporters, enzymes, and ferritin, thereby protecting the cell from excessive Reactive Oxygen Species formation (Shi et al., 2008). PCBP2 acts as a cytosolic iron chaperone, ensuring safe intracellular iron trafficking: in its apo form, it interacts with the Divalent Metal Transporter 1 to receive Fe^2+^ from endosomes, whereas in its holo form, it transfers iron to ferroportin (FPN1) for export (Yanatori et al., 2016). Furthermore, PCBP2 forms a metabolon with heme oxygenase 1 and cytochrome P450 reductase, capturing Fe^2+^ released during heme degradation and preventing iron-mediated oxidative stress (Yanatori et al., 2017). PCBP1, either alone or in cooperation with PCBP2, delivers Fe^2+^ to ferritin and participates in ferritinophagy, cooperating with Nuclear Receptor Coactivator 4 during autophagic recycling of iron (Shi et al., 2008; Ryu et al., 2017). Recent studies show that Dex disrupts iron metabolism in mouse muscle, as evidenced by an increase in the labile iron pool (Jeong et al., 2025); however, to the best of our knowledge, no data are available on the proteins involved in iron metabolism across different muscle types.

Another factor that disrupts the balance between skeletal muscle protein synthesis and breakdown is physical exercise. During endurance exercise, skeletal muscle protein synthesis is depressed (Gautsch et al., 1998), and these changes may be associated with increased AMP-activated protein kinase activity and reduced mTOR signaling (Williamson et al., 2006). After a single bout of exercise, FOXO3a protein and mRNA levels increased (Slopack et al., 2014), and FOXO3a is essential for inducing protein breakdown and autophagy (Sanchez et al., 2014). In contrast, the FoxO gene family can increase the expression of antioxidant enzymes, thereby reducing oxidative stress (Krafczyk and Klotz, 2022). Interestingly, physical training prevents GC-related muscle atrophy. Eight weeks of resistance training ameliorates Dex-induced atrophy by increasing mTOR and reducing Atrogin-1 and Muscle-specific RING finger protein 1 (MuRF-1) protein levels (Macedo et al., 2014). Physical exercise also regulates muscle iron metabolism. Results show that in muscles with iron accumulation, regular endurance training lowers iron stores, as reflected in reduced ferritin levels (Ryan et al., 2021). This beneficial change may reflect protection against oxidative stress and consequent cellular dysfunction (Reardon and Allen, 2009; Liang et al., 2019).

In contrast, intense exercise can increase iron levels after physical activity. Therefore, after this type of exercise, a protective adaptation to chelate “free” iron released from hemolysis should be observed to limit oxidative damage (Halon-Golabek et al., 2019). A similar observation was noted in the hearts after long-lasting exercise, where an elevation of the ferritin L subunit, which may be involved in storing excess labile iron, was observed (Ziolkowski et al., 2015).

Despite the protective effects of physical training on Dex-induced skeletal muscle atrophy, no data exist on the effects of Dex on skeletal muscle iron metabolism and AKT-mediated signaling after acute prolonged exercise. Also, no results have been reported on the effect of acute exercise on skeletal muscle iron metabolism-related proteins. Although Dex and exercise, administered separately, modify iron metabolism, suppress mTOR signaling, and promote catabolic pathways, it is unknown whether exercise preceded by Dex treatment will 1) enhance the effect of Dex on the AKT-dependent pathway and 2) have an identical effect on muscles with different muscle fiber compositions, resulting in changes in muscle iron handling protein levels and muscle mass. This issue appears to be important for patients undergoing Dex treatment while maintaining physical activity. Therefore, this study investigates the effects of acute prolonged swimming on skeletal muscle oxidative stress, iron metabolism, and AKT-mediated signaling in muscle from non-treated and Dex-treated rats with different fiber types (i.e., fast-twitch and slow-twitch). We hypothesize that Dex treatment induces oxidative stress, modifies muscle iron-handling proteins, suppresses mTOR signaling, and promotes catabolic pathways, depending on skeletal muscle fiber composition, and that acute prolonged swimming enhances the effect of dexamethasone.

## 2 Materials and methods

### 2.1 Animals

The 3rd Local Ethical Committee for Experiments on Animals in Gdansk approved all experimental procedures (decision no. 51/2015, issued on 14 December 2015), and they were conducted in accordance with the European Union Directive 2010/63/EU. Animals for this study were purchased from the Tri-City Academic Laboratory Animal Center. Male Wistar rats (4 groups, n = 8 per group), weighing 250-300 g, were housed in an environmentally controlled room (23 ± 1 °C with a 12 h light-dark cycle). All animals received standard rat chow and water ad libitum. After the acclimatization period, rats were randomly assigned to the following groups: SS - saline injection, sedentary group; SE - saline injection, exercise group with exercise preconditioning; DS– dexamethasone injection, sedentary group; DE - dexamethasone injection, exercise group with exercise preconditioning. Rats were weighed daily at the same time to determine both the Dex dose and the exercise load.

The animals were euthanized by decapitation, preceded by stunning by being hit on the head with a blunt instrument. Skeletal muscles: Soleus (SOL) and Extensor Digitorum Longus (EDL) were immediately removed, weighed, frozen in liquid nitrogen, and stored at -80 °C until analysis.

### 2.2 Injection of dexamethasone and saline

Dexamethasone and saline were administered by intraperitoneal injection for 9 days straight. The DS and DE groups received dexamethasone (1 mg/kg of body weight, dissolved in saline solution). The SS and SE groups received the same amount of saline solution as the DS and DE groups.

### 2.3 Swim exercise procedure

The rats were prepared for the experiments and exercise tests using methods described by Keatisuwan et al. (1991), with slight modifications (Flis et al., 2016, Flis et al., 2022). Before acute prolonged exercise, animals in the exercise groups (DE and SE) were trained to reduce the stress associated with swimming. Each day during the preparatory procedure, the rats swam for 30 min in water heated to 35 °C. On the first day, no extra load was attached to the rats while swimming. On the second, third, and fourth days, the rats swam with tail-mounted loads of 1%, 2%, and 3% of their body weight, respectively. On the fifth day, exercise consisted of 3 hours of prolonged swimming in 35 °C water while carrying a load equivalent to 3% of their body weight. Immediately after completing the protocol, we euthanized the rats. The preparatory procedure started on the 6th day of Dex treatment, the first day of significant reduction in EDL muscle mass (**Scheme 1**; **Supplementary Figures 1a, b**).

**Scheme 1:**
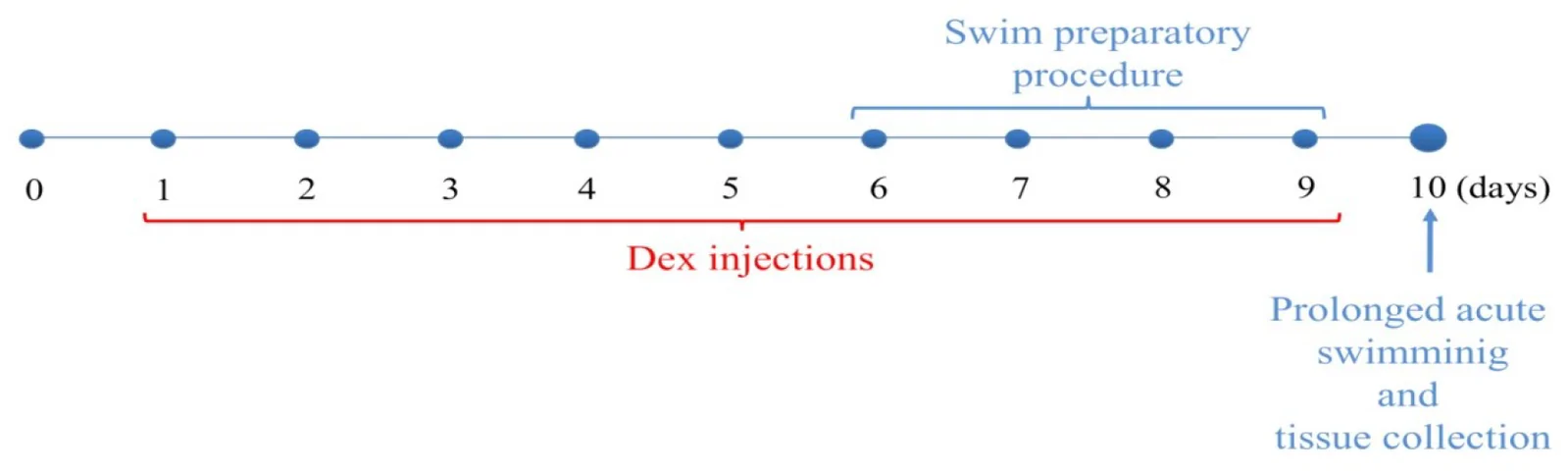
Experimental timeline.

### 2.4 Sample preparation

For Western blotting, the unfrozen muscle was weighed and immediately transferred to ice-cold RIPA lysis buffer supplemented with protease and phosphatase inhibitor cocktails. Then, a 10% tissue lysate was made using a manual glass homogenizer. The lysate was frozen and thawed three times at -80 °C and 30 °C, then homogenized once again. Finally, the homogenate was centrifuged at 15,000g for 10min at 4 °C. The resulting supernatant was collected and stored at -80 °C for further analysis.

For catalase activity and markers of free radical damage, the unfrozen muscle was weighed and immediately transferred into ice-cold buffer (50 mM Potassium Phosphate, 1 mM EDTA, 0.5 mM DTT, 1.15% KCl, pH 7.4, supplemented with protease inhibitor cocktail). After homogenization (approximately 30 strokes), the homogenate was immediately frozen and stored at –80 °C until analysis.

### 2.5 Western blotting

Equal amounts of lysate were applied to 4–20% SDS-polyacrylamide gradient gels and separated by electrophoresis. After electrophoresis was complete, the gels were UV-activated using the ChemiDoc Imaging System (Bio-Rad, USA). Then, the proteins were transferred from gels to PVDF membranes using the Trans-Blot Turbo Transfer System (Bio-Rad, USA). Once the transfer was completed, membranes were blocked in EveryBlot Blocking Buffer. Next, the membranes were incubated overnight at 4 °C with primary antibodies in containers placed on a shaker. The following primary antibodies were used: PCBP1 (Cell Signaling, Cat. #8534, 1:1000), PCBP2 (Cell Signaling, Cat. #83017, 1:1000), Ferritin L (Abcam, Cat. #ab69090, 1: 1000), Ferritin H (Cell Signaling, Cat. #3998, 1:1000), APP (Cell Signaling, Cat. #2452, 1:1000), FPN1 (Bioss, #bs-3579r, 1:1000), AKT (Cell Signaling, Cat. #4691, 1:1000), p-AKT (Ser473) (Cell Signaling, Cat. #4060, 1:1000), IGF1R (ABclonal, Cat. A21984, 1:1000), mTOR (Cell Signaling, Cat. #2983, 1:1000), p-mTOR (Ser2448) (Cell Signaling, Cat. #5536, 1:1000), p70S6K (Cell Signaling, Cat. #34475, 1:1000), p-p70S6K (Thr389) (Cell Signaling, Cat. #97596, 1:250), FOXO3a (Cell Signaling, Cat. #12829, 1:1000), p-FOXO3a (Ser253) (Cell Signaling, Cat. #13129, 1:1000), Atrogin-1 (Santa Cruz Biotechnology, Cat. sc-166806, 1:500), MuRF1 (Santa Cruz Biotechnology, Cat. sc-398608, 1:500).

After incubation with primary antibodies, the membranes were washed 5 times for 5 minutes in TBST (150 mM NaCl, 10 mM Tris, 0.1% Tween 20, pH 7.0), then incubated with secondary antibodies for 1 hour at room temperature. The following secondary antibodies were used: anti-Rabbit IgG–Peroxidase (Sigma-Aldrich, Cat. #A9169, 1:25,000) and anti-Mouse IgG–Peroxidase (Sigma-Aldrich, Cat. #A3682, 1:25,000).

After incubation with secondary antibodies, the membranes were washed in TBST once again (5 times, 5 minutes each), and immunoblots were detected and visualized using enhanced chemiluminescence reagents (Westar Supernova, Cyanagen, Italy). Changes in protein levels were assessed by densitometry of the immunoreactive bands (**Supplementary Figure 2–S18**) and normalized to the total amount of protein (as determined by the Stain-Free image, **Supplementary Figure 19**, and presented in supplementary figures **Supplementary Figures 2–S18**) transferred onto the membrane in each lane, which provides the best possibility to normalize analyzed proteins and avoid mistakes related to Housekeeping proteins. In the next step, we normalized the analyzed proteins to a single control (the same on each blot). In this way, all data were normalized to the same control animal (our 1 (100%), NORM lane in **Supplementary Figure 19**). Protein levels were analyzed and quantified using Image Lab Software (Bio-Rad, USA).

### 2.6 Measurement of catalase activity

Catalase (CAT) activity was measured spectrophotometrically at 30 °C as described by Aebi (Aebi, 1984). CAT activity was measured in a mixture containing 50 mM potassium phosphate, 5 mM EDTA, and 0.01% Triton at pH 7.4. The addition of H_2_O_2_ started the reaction. The kinetics of H_2_O_2_ decomposition were followed over time at 240 nm. Then, values were calculated and expressed as nmol/min/mg of protein.

### 2.7 Measurement of peroxidation markers

#### 2.7.1 Thiol groups

Concentration of thiol groups (SH), a biomarker of protein peroxidation, was determined according to Ellman’s method (Ellman, 1959) against the standard curve (standard–reduced glutathione). The lowest concentration of reduced glutathione in the standard curve was 0.25 mmol/L. The standard curve ranged from 0 to 2 mmol/L. In brief, 200 μL of sodium phosphate buffer (10 mM, pH 8.0), 30 μL of sodium dodecyl sulfate (10%), and 20 μL of plasma were added and mixed. Subsequently, 30 μL of DTNB (1 mM) was added to the experimental sample (prepared in duplicate) or to the reference sample containing 30 μL of sodium phosphate buffer. The samples were then incubated at 37 °C for 20 min, and absorbance was measured at 415 nm in a Synergy H1 Hybrid Multi-Mode microplate reader (BioTek, USA).

#### 2.7.2 8-isoprostanes

The concentration of 8-isoprostanes, a biomarker of lipid peroxidation, was determined with the 8-isoprostane ELISA Kit (516351, Cayman Chemicals, USA).

### 2.8 Statistical analysis

Statistical analyses were performed using the software Statistica v. 13.0 (StatSoft Inc., Tulsa, OK, USA). The results are expressed as the mean ± standard error (SEM). Differences between means in the analyzed data were tested using two-way ANOVA. If a difference was detected in the ANOVA model, the Tukey *post hoc* test was used to determine significant differences. When the two-way ANOVA analysis showed a significant interaction or main effect for Dex treatment or exercise procedure, but the Tukey *post hoc* test did not present significant differences, the least significant difference (LSD) *post hoc* test was used to determine tendencies in the results. The results were considered statistically significant when p < 0.05.

## 3 Results

### 3.1 The effect of Dex treatment and exercise procedure on rats’ skeletal muscles and body mass

There was a statistically significant interaction between Dex treatment and exercise protocol on SOL muscle mass (F(1, 32) = 16.419, p < 0.001, η^2^_p_ = 0.339). Nine days of Dex treatment significantly reduced SOL mass (102.62 ± 2.84 mg) in comparison to SS (117.44 ± 1.97 mg, p= 0.006; **Figure 1a**). The swim procedure protected muscle mass, as observed in DE groups (121.81 ± 4.14 mg; p < 0.001 compared to DS groups; **Figure 1a**).

**Figure 1:**
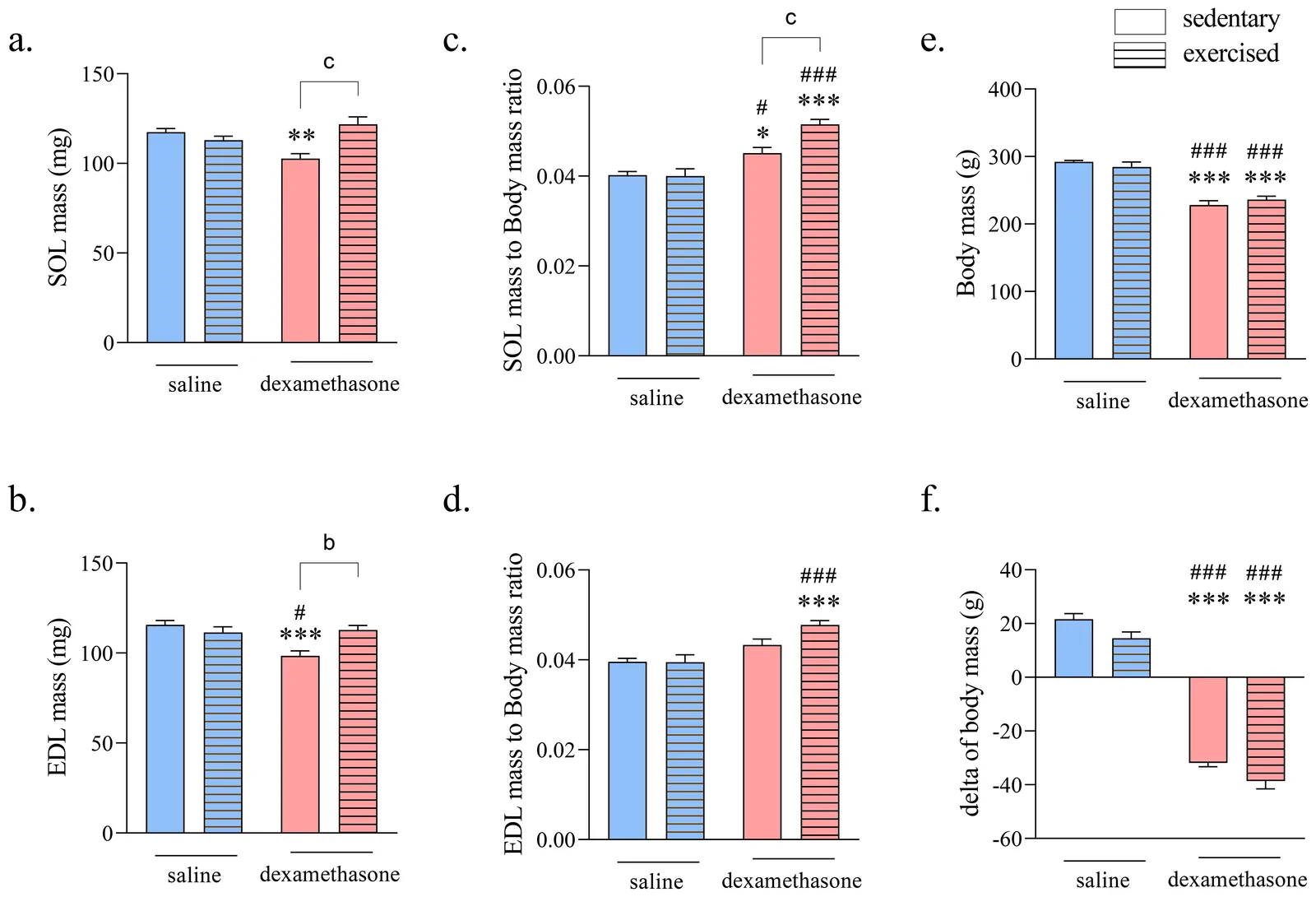
The effects of Dex treatment, swimming, and a combination of these factors on body and skeletal muscle mass. Soleus (SOL) muscle mass **(a)**, Extensor Digitorum Longus (EDL) muscle mass **(b)**; the ratio of SOL to body mass **(c)**, the ratio of EDL to body mass **(d)**; body mass **(e)**; and body mass change during the experiment **(f)** in the rat. There were significant differences between the groups: *p < 0.05, **p < 0.01, ***p < 0.001 vs. saline-sedentary group; ^#^p < 0.05, ^###^p < 0.001 vs. saline-exercised group; ^b^p < 0.01, ^c^p < 0.001 between indicated groups. The data are presented as means ± SEM (n = 8 per group).

Also, there was a statistically significant interaction between Dex treatment and exercise protocol on EDL muscle mass (F(1, 32) = 11.459, p = 0.002, η^2^_p_ = 0.264). Dex treatment significantly reduced EDL mass (98.37 ± 2.86 mg) in comparison to SS and SE groups of rats (115.69 ± 2.41 and 111.44 ± 3.15 mg, p< 0.001 and p=0.010, respectively; **Figure 1b**), and the swim procedure protected muscle mass, as observed after the swimming procedure in DE groups (112.75 mg, p = 0.004 compared to DS groups, **Figure 1b**).

There was no statistically significant interaction between Dex treatment and exercise protocol on rats’ body mass and change in body mass during the experiment; however, the main effect of Dex treatment was significant (p < 0.001 for both analyses, η^2^_p_ = 0.761 for body mass and η^2^_p_ = 0.952 for body mass delta). The main effect of exercise procedure on body mass delta was also significant (p = 0.005, η^2^_p_ = 0.250). Dex treatment significantly reduced body mass in both swimming (236.25 ± 4.82 g, - 38.63 ± 2.88 g during the experiment) and non-exercised (228.12 ± 6.31 g, - 31.88 ± 1.46 g during the experiment) animals compared to saline-treated rats (292.25 ± 1.75, + 21.62 ± 2.10 g during the experiment and 284.37 ± 7.58 g, + 14.50 ± 2.40 g during the experiment in SS and SE groups, respectively; p < 0.001, **Figures 1e, f**).

To evaluate skeletal muscle atrophy, the ratios of SOL and EDL to body mass were calculated. There was a statistically significant interaction between Dex treatment and exercise procedure on the SOL mass to body mass ratio (F(1, 32) = 7.198, p = 0.011, η^2^_p_ = 0.184). Nine days of Dex treatment significantly increase SOL to body mass ratio (0.045 ± 0.001) in comparison to SS and SE groups of rats (0.040 ± 0.001 and 0.040 ± 0.002, p= 0.037 and p= 0.029, respectively; **Figure 1c**). There were also more statistically significant changes observed after Dex treatment and exercise procedure (0.051 ± 0.001 in SOL, p< 0.001 in comparison to SS, SE, and DE groups; **Figure 1c**).

There was no statistically significant interaction between Dex treatment and exercise procedure on the EDL mass to body mass ratio; however, the main effect of Dex treatment was significant (p < 0.001, η^2^_p_ = 0.429). EDL to body mass ratio increased after Dex treatment and the swimming procedure (0.048 ± 0.001, p< 0.001) in comparison to SS and SE groups, **Figure 1d**.

### 3.2 The effect of Dex treatment and exercise on rats’ skeletal muscles’ peroxidation markers and catalase activity

To determine whether skeletal muscle atrophy is accompanied by oxidative stress, indicators of oxidative stress were measured in both examined SOL and EDL muscles. There was no statistically significant interaction between Dex treatment and exercise on SOL 8-isoprostanes level; however, the main effect of Dex treatment was significant (p < 0.001, η^2^_p_ = 0.366). Dex treatment significantly increase the 8-isoprostanes level in swimming (2026.07 ± 76.82 pg/mg of protein) animals compared to saline-treated, sedentary (1663.31 ± 40.65 pg/mg of protein, p = 0.003) and swimming rats (1684.93 ± 81.84 pg/mg of protein, p = 0.005; **Figure 2a**). There were no changes in any of the analyzed groups in the EDL muscles 8-isoprostanes concentration (**Figure 2b**).

**Figure 2:**
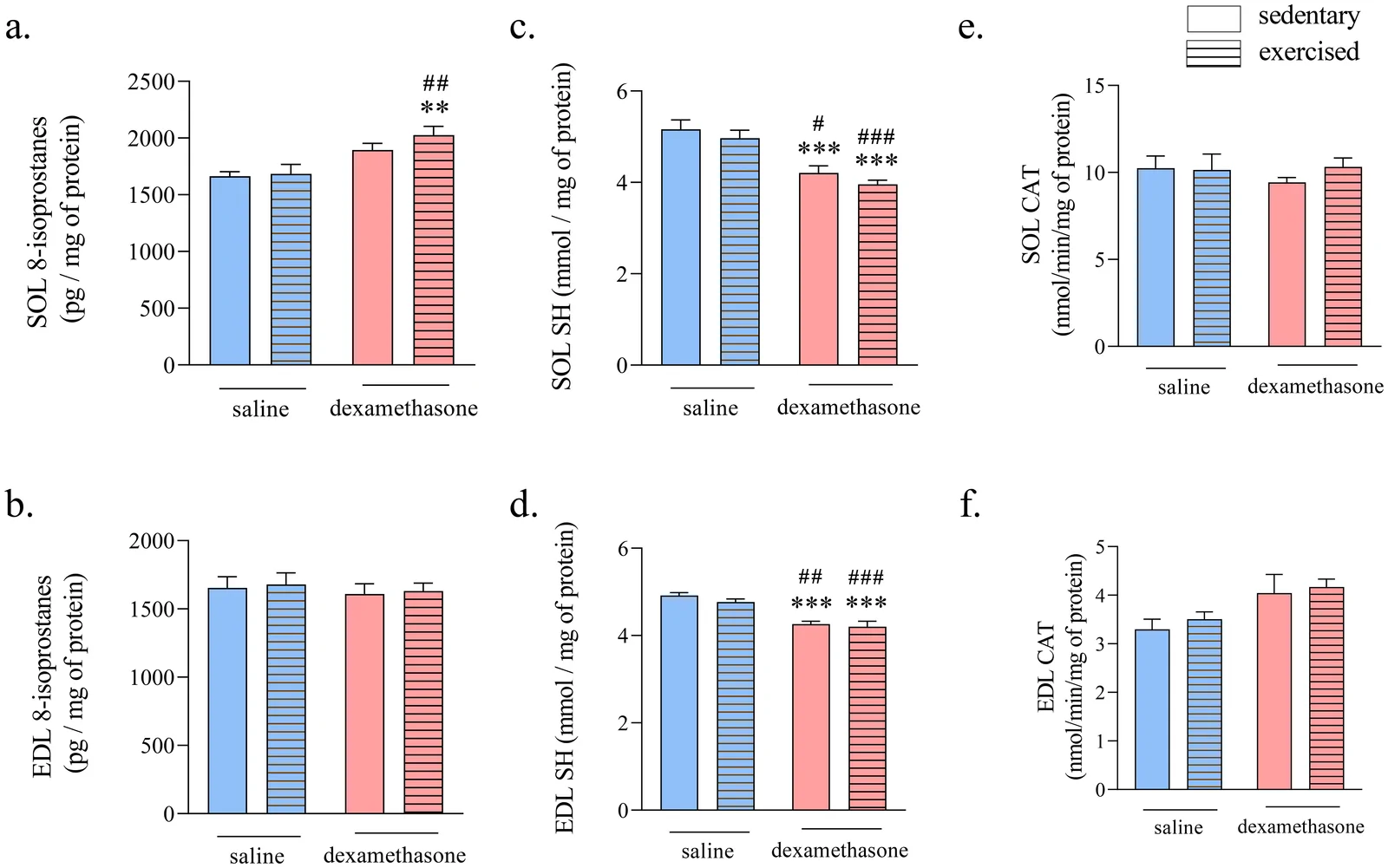
The effects of Dex treatment, swimming, and a combination of these factors on lipid and protein peroxidation markers and catalase activity in rat Soleus (SOL) and Extensor Digitorum Longus (EDL) skeletal muscles. 8-isoprostanes in SOL **(a)** and EDL **(b)**; thiol groups (SH) in SOL **(c)** and EDL **(d)**; and catalase activity (CAT) in SOL **(e)** and EDL **(f)**. There were significant differences between the groups: **p < 0.01, ***p < 0.001 vs. saline-sedentary group; ^#^p < 0.05, ^##^p < 0.01, ^###^p < 0.001 vs. saline-exercised group. The data are presented as means ± SEM (n = 8 per group).

There was no statistically significant interaction between Dex treatment and exercise on SOL and EDL SH concentration; however, the main effect of Dex treatment was significant (p < 0.001 for both muscles, η^2^_p_ = 0.535 for SOL and η^2^_p_ = 0.621). In SOL muscle Dex treatment significantly reduced SH concentration in both sedentary (4.21 ± 0.15 mmol/mg of protein) and exercised (3.95 ± 0.09 mmol/mg of protein) animals, in comparison to saline-treated rats (5.16 ± 0.20 and 4.96 ± 0.18 mmol/mg of protein in SS and SE groups, respectively, p= 0.001 for DS vs. SS, p= 0.012 for DS vs. SE, and p< 0.001 for rest comparisons; **Figure 2c**). In EDL muscle Dex treatment significantly reduced SH concentration in both sedentary (4.26 ± 0.06 mmol/mg of protein) and exercised (4.20 ± 0.12 mmol/mg of protein) animals, in comparison to saline-treated rats (4.92 ± 0.06 and 4.76 ± 0.07 mmol/mg of protein in SS and SE groups, respectively, p= 0.001 for DS vs. SE p< 0.001 for all comparisons; **Figure 2d**).

There were no changes in any of the analyzed groups in the SOL muscles’ CAT activity (**Figure 2e**). There was no statistically significant interaction between Dex treatment and exercise on EDL CAT activity; however, the main effect of Dex treatment was significant (p = 0.007, η^2^_p_ = 0.205). In EDL muscle there is a tendency to increase the CAT activity in Dex treatment sedentary (4.04 ± 0.38 nmol/min/mg of protein) and swimming (4.17 ± 0.16 nmol/min/mg of protein) animals compared to saline-treated, sedentary rats [3.30 ± 0.21 nmol/min/mg of protein, p= 0.039 and p= 0.017 for DS and DE groups (LSD *post hoc* test); **Figure 2f**]. There was almost three times higher CAT activity in SOL than in EDL (p< 0.001).

### 3.3 The effect of Dex treatment and acute prolonged swimming on rats’ skeletal muscles’ iron storage, intracellular trafficking, and export proteins

To check whether oxidative stress is iron-dependent, proteins responsible for iron metabolism were measured in both SOL and EDL muscles. There was no statistically significant interaction between Dex treatment and acute prolonged swimming on FH levels in both muscles; however, the main effect of Dex treatment was significant (p = 0.001, η^2^_p_ = 0.307 for SOL and p = 0.007, η^2^_p_ = 0.234 for EDL). In the SOL muscle, nine days of Dex treatment significantly increase FH levels in sedentary (3.76 ± 0.62 A.U.) and swimming (3.52 ± 0.38 A.U.) animals compared to the SS group (2.09 ± 0.23 A.U., p = 0.014 and p = 0.042, respectively; **Figure 3a**). In the EDL muscle, there was a significant increase in FH level in the DS group (1.18 ± 0.19 A.U.) compared to SS (0.66 ± 0.08 A.U., p = 0.038; **Figure 3b**). There was also a significantly higher level of FH in SOL than in EDL muscle groups (p<0.01).

**Figure 3:**
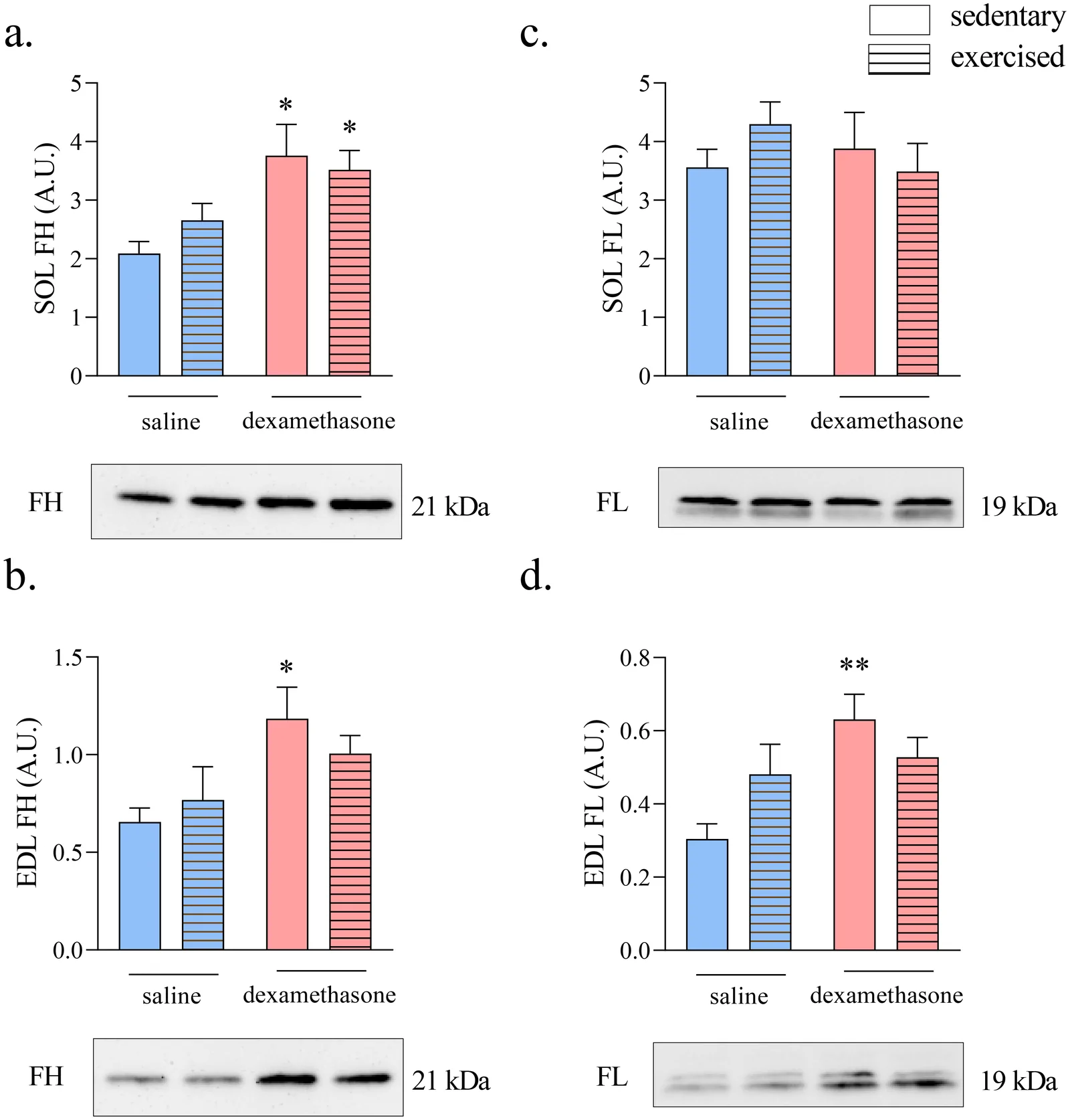
The effects of Dex treatment, swimming, and a combination of these factors on iron storage proteins in rat Soleus (SOL) and Extensor Digitorum Longus (EDL) skeletal muscles. Ferritin H (FH) in SOL **(a)** and EDL **(b)**; Ferritin L (FL) in SOL **(c)** and EDL **(d)**. There were significant differences between the groups: *p < 0.05, **p < 0.01 vs. saline-sedentary group. The data are presented as means ± SEM (n = 7 in **(a, b)** and n = 5 in **(c, d)**.

There were no changes in FL level after Dex treatment and acute prolonged swimming in any of the groups in the SOL muscle (**Figure 3c**). There was a statistically significant interaction between Dex treatment and acute prolonged swimming on EDL FL level (F(1, 20) = 4.905, p = 0.038, η^2^_p_ = 0.197). The FL protein level increases after Dex treatment in EDL muscle (0.63 ± 0.08 A.U.) compared to the SS group (0.30 ± 0.05 A.U., p = 0.008; **Figure 3d**). There were also almost six times higher FL levels in SOL than in EDL (p<0.001).

There was a statistically significant interaction between Dex treatment and acute prolonged swimming on SOL PCBP1 protein level (F(1, 20) = 5.835, p = 0.025, η^2^_p_ = 0.226). Dex treatment significantly reduced the SOL PCBP1 protein level in both swimming (2.35 ± 0.61 A.U.) and non-exercised (2.87 ± 0.65 A.U.) animals compared to saline-treated, sedentary rats (6.81 ± 0.44 A.U., p<0.001 for both groups; **Figure 4a**). Also, a significant decrease in PCBP1 level was observed after acute prolonged swimming in SE group (4.09 ± 0.51 A.U., p = 0.002; **Figure 4a**). In EDL muscle, there was no statistically significant interaction between Dex treatment and acute prolonged swimming on PCBP1 protein level; however, the main effect of Dex treatment was significant (p = 0.031, η^2^_p_ = 0.213). There was a tendency toward a reduction in EDL PCBP1 level after Dex treatment (0.10 ± 0.01 A.U.) in comparison to the SS group [0.15 ± 0.02 A.U., p = 0.047 (LSD *post hoc* test); **Figure 4b**]. There was also at least fifteen times higher PCBP1 level in SOL than in EDL (p < 0.001).

**Figure 4:**
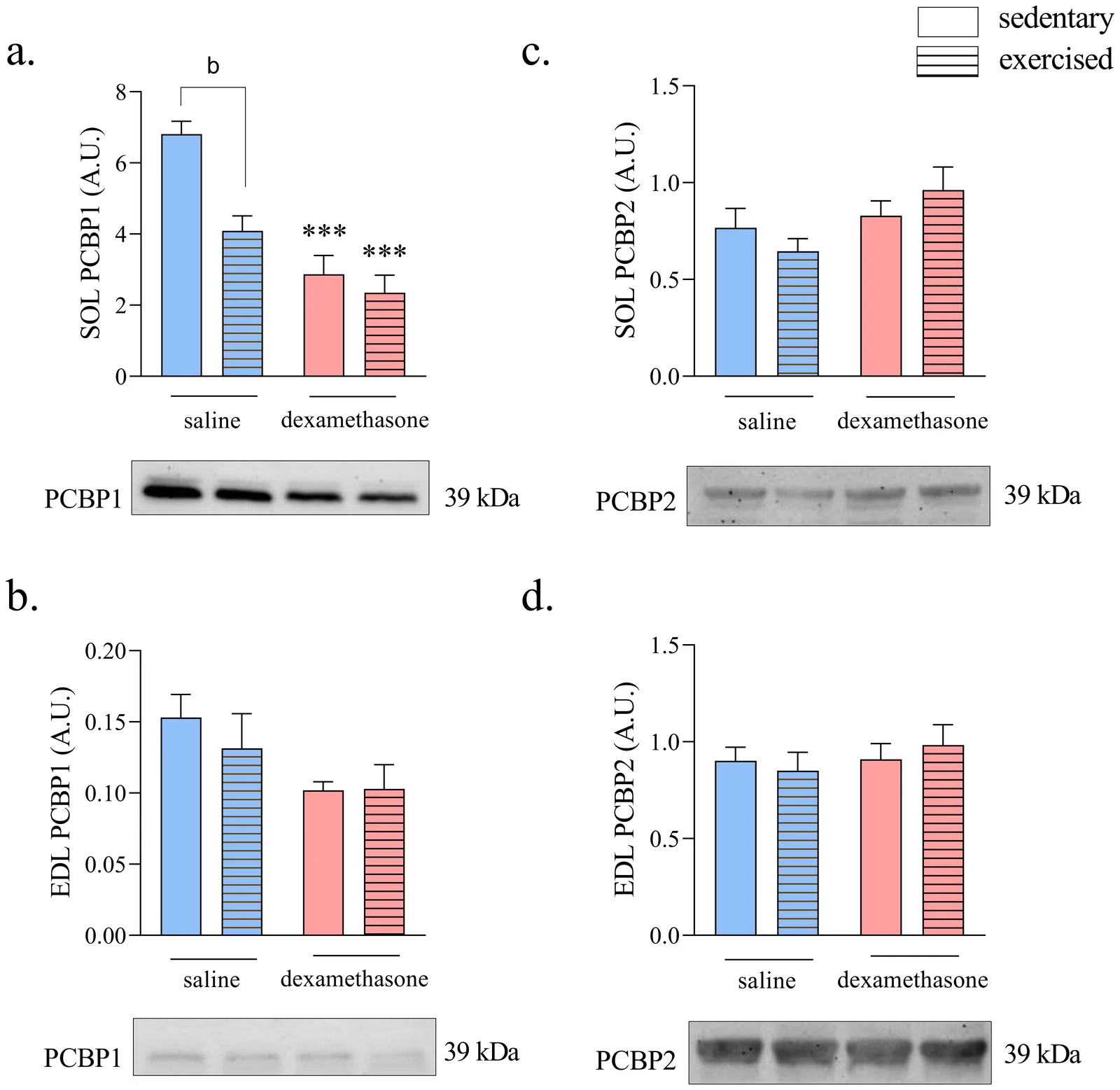
The effects of Dex treatment, swimming, and a combination of these factors on iron intracellular transport proteins in rat Soleus (SOL) and Extensor Digitorum Longus (EDL) skeletal muscles. Poly(rC)-Binding Protein 1 (PCBP1) in SOL **(a)** and EDL **(b)**; Poly(rC)-Binding Protein 2 (PCBP2) in SOL **(c)** and EDL **(d)**. There were significant differences between the groups: ***p < 0.001 vs. saline-sedentary group; ^b^p < 0.01 between indicated groups. The data are presented as means ± SEM (n = 5 in each group **(a, b)**, n = 7 in **(c)**, and n=6 in **(d)**.

There were no changes in the SOL and EDL PCBP2 protein levels across any of the analyzed groups (**Figures 4c, d**).

There were no changes in FPN1 and APP protein levels in the SOL and EDL muscles across the analyzed groups (**Supplementary Figures 20a–d**); however, FPN1 levels were almost three times higher in SOL than in EDL (p < 0.001), and APP levels were also almost four times higher in SOL than in EDL (p<0.001).

### 3.4 The effect of Dex treatment and acute prolonged swimming on the rats’ skeletal muscles’ Akt signaling pathway

#### 3.4.1 IGF-1/Akt signaling pathway proteins

As previously mentioned, Dex-induced skeletal muscle atrophy may affect the AKT signaling pathway. Therefore, we measured proteins involved in later stages of this pathway, including those involved in the pathways associated with synthesis and proteolysis of skeletal muscle proteins.

There was no statistically significant interaction between Dex treatment and acute prolonged swimming on Insulin-like Growth Factor 1 Receptor (IGF1R) protein level; however, the main effect of Dex treatment was significant (p = 0.038, η^2^_p_ = 0.128). The IGF1R protein level significantly decreased after Dex treatment in the DS group (0.68 ± 0.07 A.U.) compared to the SE group (0.096 ± 0.06 A.U., p = 0.033; **Supplementary Figure 21a**). There were no changes in IGF1R protein levels in any of the analyzed groups in the EDL muscles (**Supplementary Figure 21b**).

There was a statistically significant interaction between Dex treatment and acute prolonged swimming on SOL AKT levels (F(1, 32) = 9.884, p = 0.004, η^2^_p_ = 0.236). In the SOL muscle, swimming in saline-treated rats results in a decrease in AKT levels (0.75 ± 0.06 A.U., p = 0.015 vs. SS group; **Figure 5a**). There were no changes in AKT protein levels in any of the analyzed groups in the EDL muscle (**Figure 5b**).

**Figure 5:**
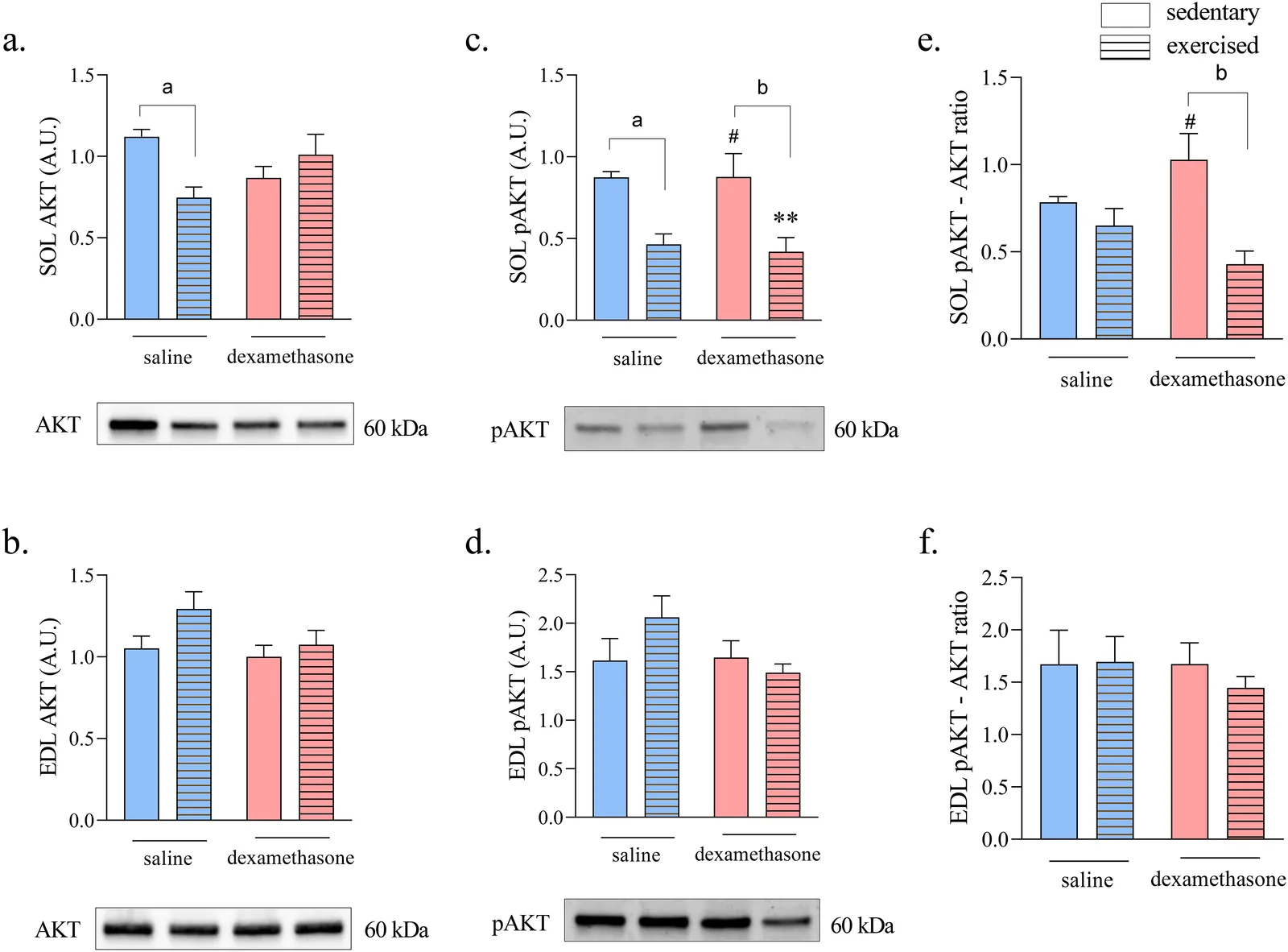
The effects of Dex treatment, swimming, and a combination of these factors on protein kinase B (AKT) in rat Soleus (SOL) and Extensor Digitorum Longus (EDL) skeletal muscles. Total AKT in SOL **(a)** and EDL **(b)**; active form of AKT (p-AKT (Ser 473)) in SOL **(c)** and EDL **(d)**; and p-AKT to AKT ratio in SOL **(e)** and EDL **(f)**. There were significant differences between the groups: **p < 0.01 vs. saline-sedentary group; ^#^p < 0.05 vs. saline-exercised group; ^a^p < 0.05, ^b^p < 0.01 between indicated groups. The data are presented as means ± SEM (n = 8 per group).

In the SOL muscle, there was no statistically significant interaction between Dex treatment and acute prolonged swimming on active AKT form (pAKT) level; however, the main effect of acute prolonged swimming was significant (p < 0.001, η^2^_p_ = 0.413). There were lowering pAKT level after swimming in SE (0.46 ± 0.06 A.U., p=0.017) and DE (0.42 ± 0.09 A.U., p= 0.007) in comparison to sedentary groups (SS: 0.87 ± 0.04, DS: 0.88 ± 0.14, and p= 0.016 for SE vs. DS, and p= 0.007 for DE vs SS, **Figure 5c**). There were no changes in pAKT protein levels in any of the analyzed groups in the EDL muscle (**Figure 5d**).

There was a statistically significant interaction between Dex treatment and acute prolonged swimming on the SOL pAKT/AKT ratio, F(1, 32) = 5.769, p = 0.022, η^2^_p_ = 0.153. In the SOL muscle, the pAKT to AKT ratio was significantly lower after the swimming procedure in Dex-treated rats (0.43 ± 0.08) in comparison to DS (p< 0.001, **Figure 5e**). There were no changes in any of the analyzed groups in the EDL muscle pAKT to AKT ratio (**Figure 5f**).

#### 3.4.2 Proteins involved in the signaling pathway associated with proteolysis of muscle proteins

In the SOL muscle, there was a statistically significant interaction between Dex treatment and acute prolonged swimming on FOXO3a protein level (F(1, 32) = 4.748, p = 0.037, η^2^_p_ = 0.129). FOXO3a protein levels significantly increased in DE (1.75 ± 0.22 A.U.) compared to the SE group (0.94 ± 0.14 A.U., p = 0.003, **Figure 6a**). In the EDL muscle, there was no statistically significant interaction between Dex treatment and acute prolonged swimming on FOXO3a protein level; however, the main effect of acute prolonged swimming was significant (p = 0.047, η^2^_p_ = 0.117). EDL FOXO3a protein levels trended to increase in the DE group (1.51 ± 0.14 A.U.) compared to the SS group (1.07 ± 0.03 A.U., p = 0.013 (LSD *post-hoc* test), **Figure 6b**).

**Figure 6:**
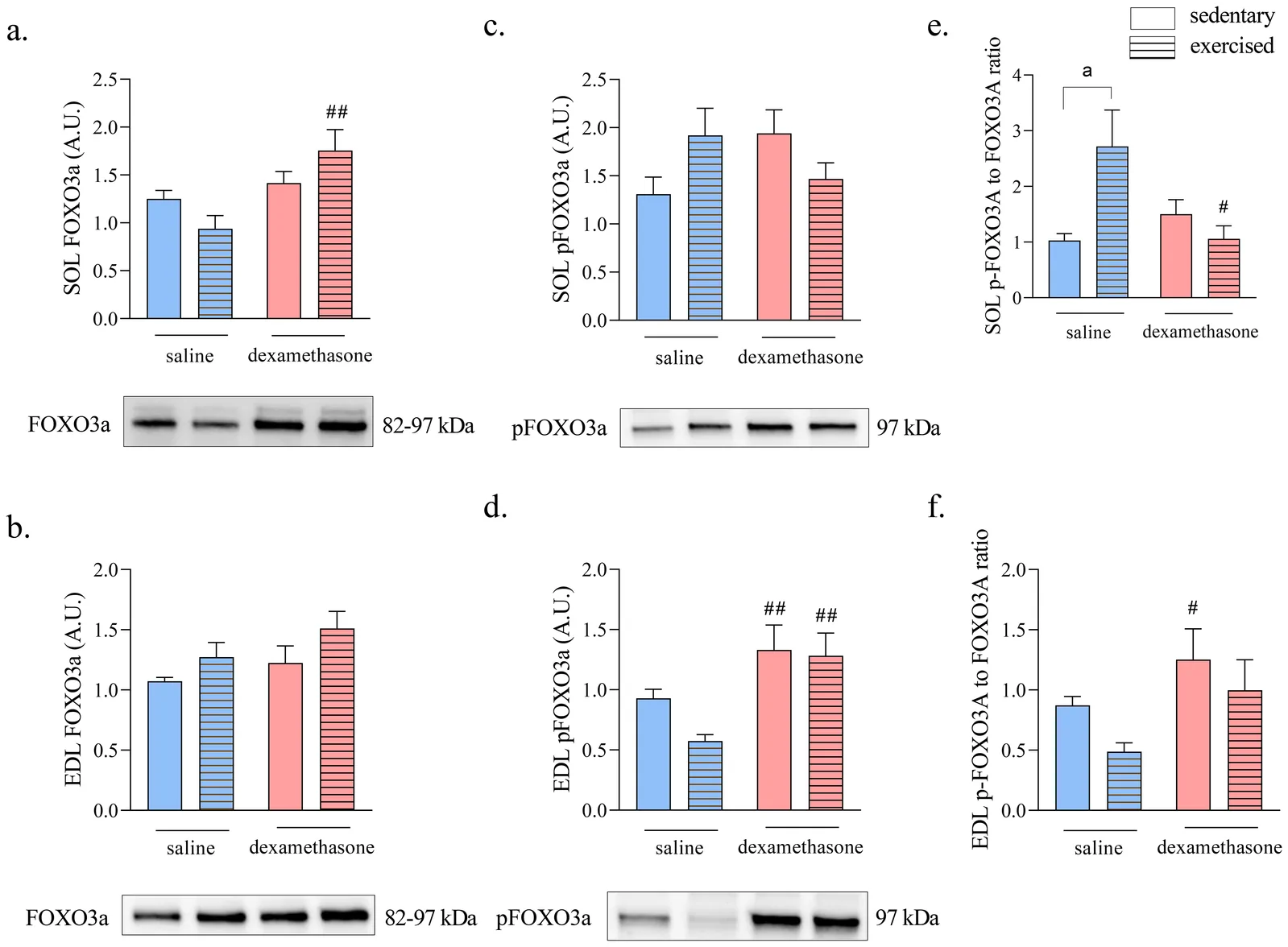
The effects of Dex treatment, swimming, and a combination of these factors on the Forkhead box O3 (FOXO3a) in rat Soleus (SOL) and Extensor Digitorum Longus (EDL) skeletal muscles. Total FOXO3a in SOL **(a)** and EDL **(b)**; inactive form of FOXO3a (p-FOXO3a (Ser253)) in SOL **(c)** and EDL **(d)**; and p-FOXO3a to FOXO3a ratio in SOL **(e)** and EDL **(f)**. There were significant differences between the groups: ^#^p < 0.05, ^##^p < 0.01 vs. saline-exercised group; ^a^p < 0.05 between indicated groups. The data are presented as means ± SEM (n = 8 per group).

There were no changes in any of the analyzed groups in the SOL muscles’ inactive form of FOXO3a (pFOXO3a, **Figure 6c**). In the EDL muscle, there was no statistically significant interaction between Dex treatment and acute prolonged swimming on pFOXO3a protein level; however, the main effect of Dex treatment was significant (p < 0.001, η^2^_p_ = 0.308). EDL pFOXO3a protein level significantly increased in the DS (1.33 ± 0.21 A.U.) and DE group (1.28 ± 0.19 A.U.) in comparison to the SE group (0.57 ± 0.05 A.U., p = 0.005 for DS and p = 0.009 for DE, **Figure 6d**).

In the SOL muscle, there was a statistically significant interaction between Dex treatment and acute prolonged swimming on the pFOXO3a to FOXO3a ratio (F(1, 32) = 7.495, p = 0.010, η^2^_p_ = 0.190). The pFOXO3a to FOXO3a ratio significantly decreases in DE (1.03 ± 0.23) compared to the SE group (2.62 ± 0.66, p = 0.025). Acute prolonged swimming in saline-treated rats induces an increase in the pFOXO3a to FOXO3a ratio in comparison to the SS group (1.03 ± 0.12, p = 0.024, **Figure 6e**). In the EDL muscle, there was no statistically significant interaction between Dex treatment and acute prolonged swimming in pFOXO3a to FOXO3a ratio; however, the main effect of Dex treatment was significant (p = 0.029, η^2^_p_ = 0.141). EDL pFOXO3a to FOXO3a ratio significantly increased in DS (1.23 ± 0.25) in comparison to the SE group (0.48 ± 0.07, p = 0.038, **Figure 6f**).

In the SOL muscle, there was no statistically significant interaction between Dex treatment and acute prolonged swimming on Atrogin-1 protein level; however, the main effect of acute prolonged swimming was significant (p = 0.002, η^2^_p_ = 0.258). Acute prolonged swimming in saline-treated rats induced an increase in SOL Atrogin-1 protein level compared to the SS group (p = 0.003, **Figure 7a**). There were no changes in the Atrogin-1 protein level in any of the analyzed groups in the EDL muscles (**Figure 7b**). However, there was almost three times higher Atrogin-1 level in EDL than in SOL (p< 0.001).

**Figure 7:**
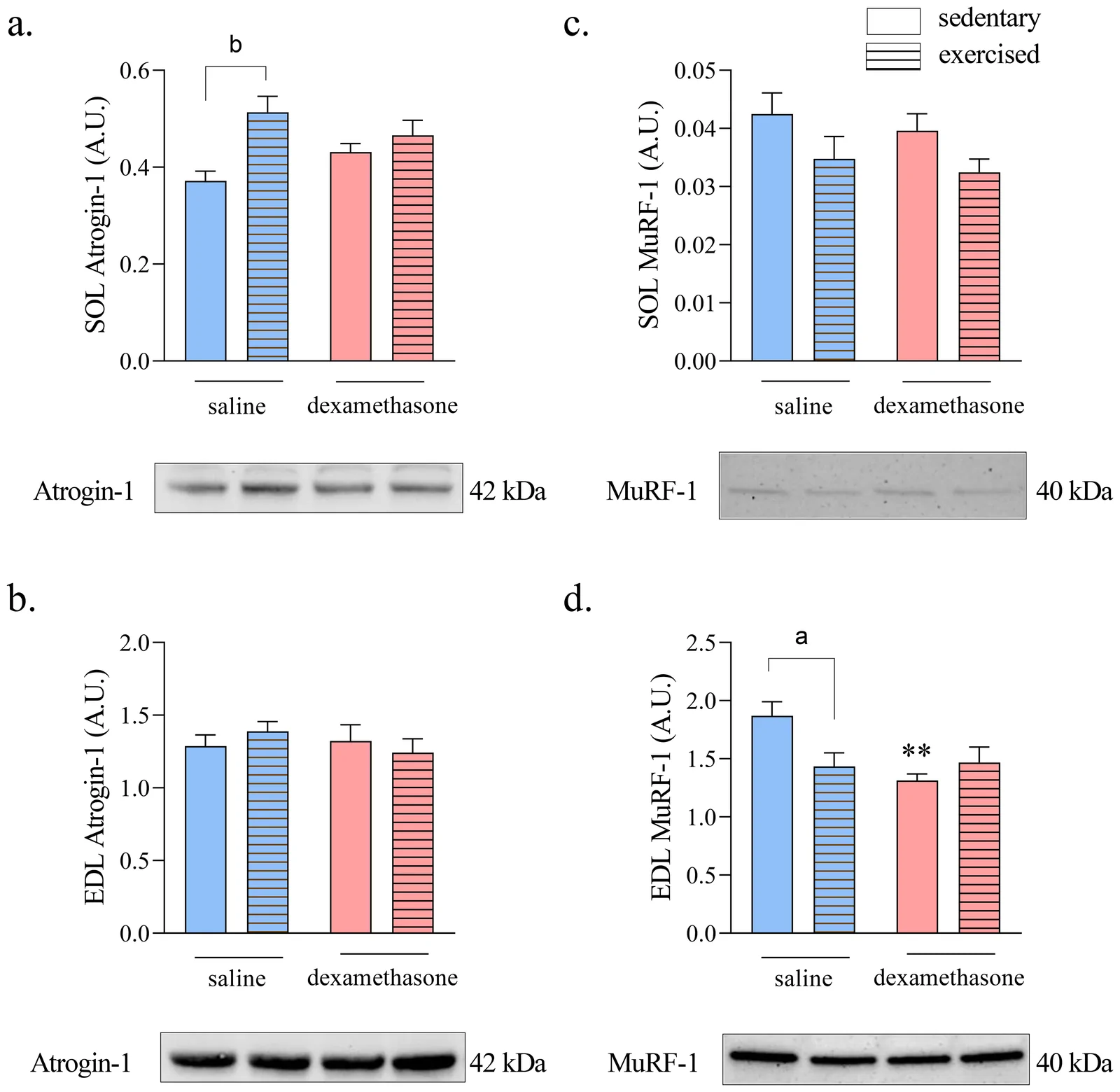
The effects of Dex treatment, swimming, and a combination of these factors on the proteins responsible for the proteolysis of muscle proteins in rat Soleus (SOL) and Extensor Digitorum Longus (EDL) skeletal muscles. Atrogin-1 in SOL **(a)** and EDL **(b)**; and Muscle RING Finger 1 (MuRF-1) in SOL **(c)** and EDL **(d)**. There were significant differences between the groups: **p < 0.01 vs. saline-sedentary group; ^a^p < 0.05, ^b^p < 0.01 between indicated groups. The data are presented as means ± SEM (n = 8 per group).

There was no statistically significant interaction between Dex treatment and acute prolonged swimming on SOL MuRF-1 protein level; however, the main effect of acute prolonged swimming was significant (p = 0.027, η^2^_p_ = 0.143). In the SOL muscle, MuRF-1 protein level trend to decreases in DE (0.03 ± 0.002 A.U.) in comparison to SS group (0.04 ± 0.004 A.U., p= 0.034 (LSD *post-hoc* test), **Figure 7c**). In the EDL muscle, there was a statistically significant interaction between Dex treatment and acute prolonged swimming on the MuRF-1 protein level, F(1, 32) = 7.066, p = 0.012, η^2^_p_ = 0.180. MuRF-1 protein level significantly decreases in EDL DS (1.31 ± 0.06 A.U.) in comparison to the SS group (1.87 ± 0.12 A.U., p = 0.007). Acute prolonged swimming in saline-treated rats also induced a decrease in EDL MuRF-1 protein level (1.43 ± 0.12, p = 0.043, **Figure 7d**) in comparison to the SS group. There was almost thirty times higher MuRF-1 level in EDL than in SOL (p< 0.001).

#### 3.4.3 Proteins involved in the signaling pathway associated with synthesis of muscle proteins

There was no statistically significant interaction between Dex treatment and acute prolonged swimming on SOL mTOR protein level; however, the main effect of Dex treatment was significant (p = 0.001, η^2^_p_ = 0.284). In the SOL muscle, the mTOR protein level significantly increases after Dex treatment in DS (1.48 ± 0.14 A.U.) and DE (1.58 ± 0.07 A.U.) groups compared to the SE group (0.92 ± 0.12 A.U., p = 0.012 for DS and p = 0.003 for DE, **Figure 8a**). In the EDL muscle, there was a statistically significant interaction between Dex treatment and acute prolonged swimming on the mTOR protein level (F(1, 32) = 5.140, p = 0.030, η^2^_p_ = 0.139). mTOR protein level significantly decreases after Dex treatment in the DS (1.01 ± 0.05 A.U.) group in comparison to the SS group (1.32 ± 0.08 A.U., p = 0.027, **Figure 8b**).

**Figure 8:**
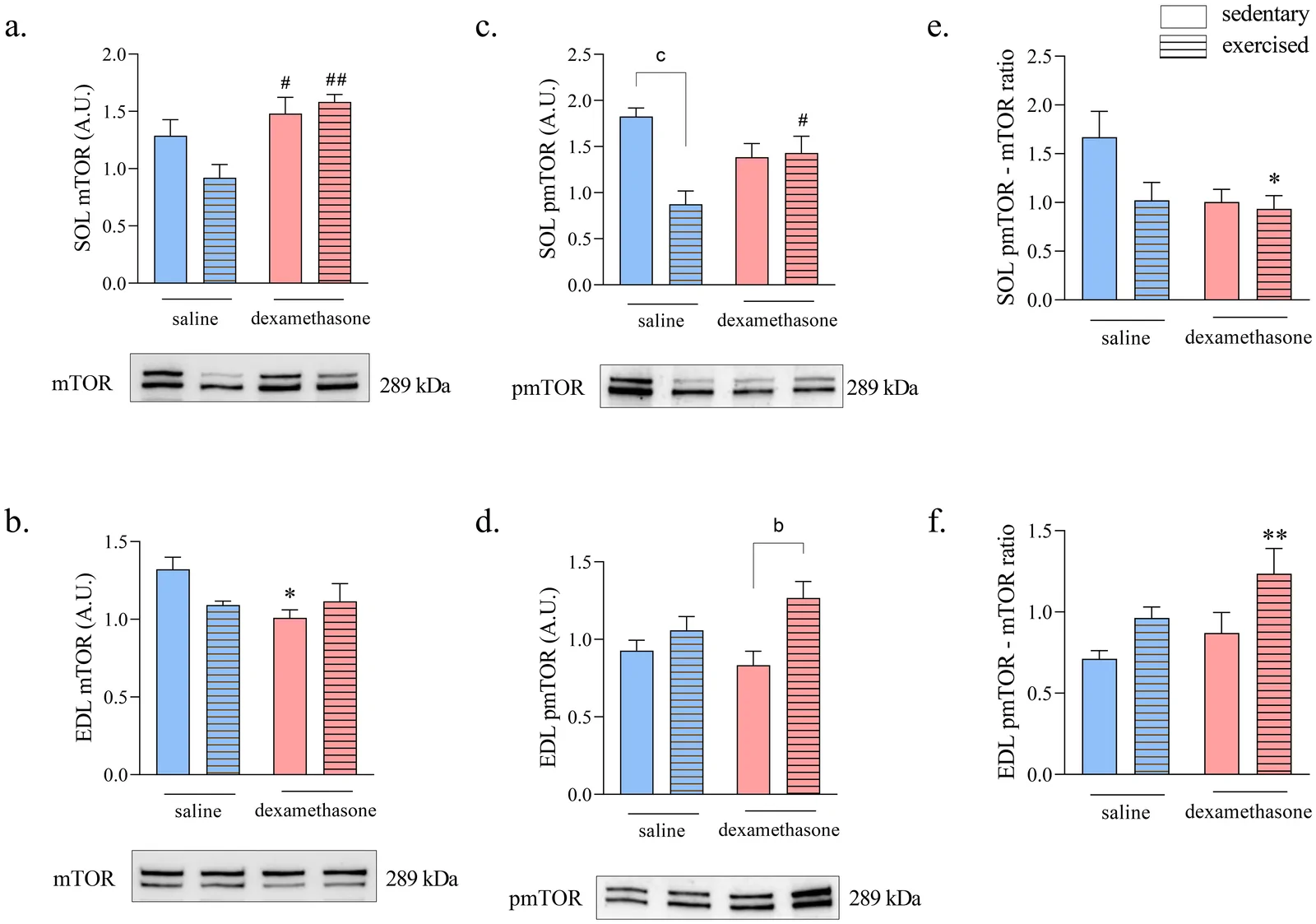
The effects of Dex treatment, swimming, and a combination of these factors on the mammalian target of rapamycin (mTOR) in rat Soleus (SOL) and Extensor Digitorum Longus (EDL) skeletal muscles. Total mTOR in SOL **(a)** and EDL **(b)**; active form of mTOR (p-mTOR (Ser2448)) in SOL **(c)** and EDL **(d)**; and p-mTOR to mTOR ratio in SOL **(e)** and EDL **(f)**. There were significant differences between the groups: *p < 0.05, **p < 0.01 vs. saline-sedentary group; ^#^p < 0.05, ^##^p < 0.01 vs. saline-exercised group; ^b^p < 0.01, ^c^p < 0.001 between indicated groups. The data are presented as the means ± SEM (n = 8 per group).

In the SOL muscle, there was a statistically significant interaction between Dex treatment and acute prolonged swimming on the active form of mTOR (pmTOR) protein level (F(1, 32) = 11.957, p = 0.002, η^2^_p_ = 0.272). The pmTOR protein level significantly increased in the Dex-treated groups (DE: 1.43 ± 0.18 A.U.) compared with the SE group (0.87 ± 0.14 A.U.; p = 0.049). Acute prolonged swimming in saline-treated rats induces a decrease in pmTOR level in comparison to the SS group (p< 0.001, **Figure 8c**). There was no statistically significant interaction between Dex treatment and acute prolonged swimming on EDL pmTOR protein level; however, the main effect of acute prolonged swimming was significant (p = 0.003, η^2^_p_ = 0.238). In the EDL muscle, acute prolonged swimming resulted in a significant increase in pmTOR protein level in the DE group (1.27 ± 0.11 A.U.) in comparison to DS (0.83 ± 0.09 A.U., p = 0.009, **Figure 8d**).

There was no statistically significant interaction between Dex treatment and acute prolonged swimming on the pmTOR to mTOR ratio in both muscles; however, the main effect of acute prolonged swimming was significant (p = 0.032, η^2^_p_ = 0.136 for SOL and p = 0.008, η^2^_p_ = 0.202 for EDL). In the SOL muscle, acute prolonged swimming in Dex-treated rats significantly decreases the pmTOR to mTOR ratio (DE: 0.92 ± 0.12) compared to the SS group (1.67 ± 0.28, p = 0.029 for DE). The pmTOR to mTOR ratio was also significantly lower after swimming in SE (0.95 ± 0.14) in comparison to SS (p= 0.037, **Figure 8e**). In EDL muscle, acute prolonged swimming during Dex treatment significantly increased the pmTOR to mTOR ratio (1.23 ± 0.15) in comparison to SS (0.71 ± 0.05, p= 0.009, **Figure 8f**).

In the SOL muscle, there was a statistically significant interaction between Dex treatment and acute prolonged swimming on p70S6K protein levels (F(1, 32) = 11.656, p = 0.002, η^2^_p_ = 0.267). The p70S6K protein level significantly increased after the acute prolonged swimming combined with Dex treatment (1.55 ± 0.15 A.U.) compared to the DE group (0.60 ± 0.07 A.U., p < 0.001, **Figure 9a**). No changes were observed in the p70S6K protein level in any of the analyzed groups in the EDL muscles (**Figure 9b**).

**Figure 9:**
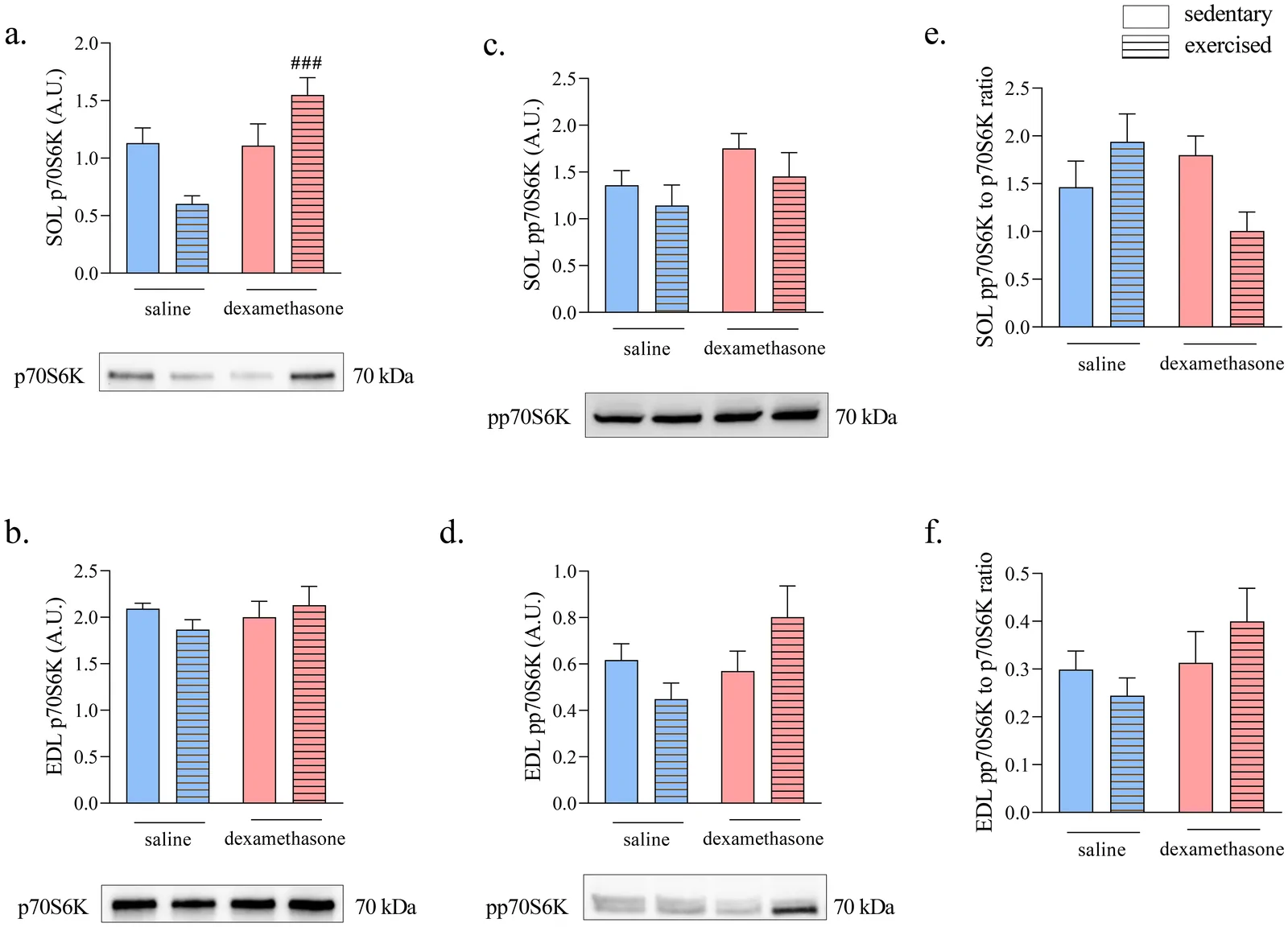
The effects of Dex treatment, swimming, and a combination of these factors on the p70 ribosomal S6 kinase (p70S6K) in rat Soleus (SOL) and Extensor Digitorum Longus (EDL) skeletal muscles. Total p70S6K in SOL **(a)** and EDL **(b)**; active form of p70S6K (p-p70S6K (Thr389)) in SOL **(c)** and EDL **(d)**; and p-p70S6K to p70S6K ratio in SOL **(E)** and EDL **(F)**. There were significant differences between the groups: ^###^p < 0.001 vs. the saline-exercised group. The data are presented as means ± SEM (n = 8 per group).

There were no changes in the active form of p70S6K (pp70S6K) protein level in any of the analyzed groups in the SOL muscles (**Figure 9c**). In the EDL muscle, there was a statistically significant interaction between Dex treatment and acute prolonged swimming on pp70S6K protein levels (F(1, 32) = 4.552, p = 0.041, η^2^_p_ = 0.124). The pp70S6K protein level trended to increase after Dex treatment combined with acute prolonged swimming (0.80 ± 0.13 A.U.) in comparison to the SE group (0.45 ± 0.07 A.U., p = 0.012 (LSD *post hoc* test), **Figure 9d**).

There was a statistically significant interaction between Dex treatment and acute prolonged swimming on the SOL pp70S6K to p70S6K ratio (F(1, 32) = 6.830, p = 0.013, η^2^_p_ = 0.176). In the SOL muscle, the pp70S6K to p70S6K ratio trend to decreased after acute prolonged swimming combined with Dex treatment (1.00 ± 0.20) in comparison to the DS group (1.77 ± 0.20, p = 0.032 (LSD *post-hoc* test)) and SE (1.93 ± 0.29, p = 0.011 (LSD *post-hoc* test), **Figure 9e**). There were no changes in any of the analyzed groups in the EDL muscles pp70S6K to p70S6K ratio (**Figure 9f**).

## 4 Discussion

In the present study, we found that 9 days of Dex treatment decreased SOL and EDL skeletal muscle mass. These harmful conditions were accompanied by increased protein and lipid peroxidation, which may be related to the disturbed iron metabolism observed after Dex injection. Previously, we have shown that an AKT-dependent pathway controls iron metabolism (Halon-Golabek et al., 2018, Halon-Golabek et al., 2024), which also controls signaling pathways associated with skeletal muscle protein synthesis and proteolysis (Scicchitano et al., 2018). In this study, we demonstrated that Dex treatment affected AKT-related pathways, thereby potentially regulating skeletal muscle mass-associated signaling pathways. Contrary to the hypothesis, our results indicate that Dex-induced loss of muscle mass is lower after a 4-day preparatory exercise procedure, after which acute prolonged exercise was performed. Also, acute prolonged swimming in Dex-treated rats modified signaling pathways involved in skeletal muscle synthesis and proteolysis, as well as proteins related to iron metabolism. Interestingly, differences between muscle types in the proteolysis/synthesis signaling pathway highlight contrasting responses.

### 4.1 The effect of Dex treatment on skeletal muscle mass, oxidative stress, iron metabolism, and protein synthesis and proteolysis pathways

Muscle atrophy is a profound side effect of synthetic GCs (Ahtikoski et al., 2004; Barel et al., 2010; Macedo et al., 2014). Dex primarily acts on muscles, with a predominance of type IIb fast-twitch fibers, such as EDL, compared with type I slow-twitch fibers, such as SOL (Barel et al., 2010; Macedo et al., 2014). These may be related to higher levels of Atrogin-1 and MuRF-1 in EDL than in SOL muscles, and higher levels of proteins related to iron metabolism (FH, FL, PCBP1, APP, and FPN-1) in SOL than in EDL, which, together with higher activity of CAT, leads to oxidative stress prevention. In this study, we also observed a reduction in muscle mass in both SOL and EDL. Similar muscle atrophy in both types of muscles was observed in untrained animals by Ahtikoski et al. (2004) and Jhuo et al. (2023), and this may be related to the animals’ age at the beginning of Dex treatment and a sedentary lifestyle (Ahtikoski et al., 2004; Jhuo et al., 2023).

One factor linked to muscle atrophy caused by various conditions, such as GC treatment (Ulla et al., 2022), denervation (Muller et al., 2007), neurodegenerative disease (Halon et al., 2014), or disruption of iron metabolism (Ikeda et al., 2016), is oxidative stress. In this study, we confirmed the relationship between Dex-induced muscle atrophy, oxidative stress, and disorders of iron metabolism. In our previous study, we observed increased oxidative stress markers and AKT-dependent increases in FH and FL protein levels, indicating increased muscle iron storage (Halon-Golabek et al., 2018). Jeong et al. (2025) demonstrated that exposure of muscle cells to Dex increases the labile iron pool (LIP). Elevated LIP, which may contribute to the Dex-induced rise in oxidative stress, activates the Iron Regulatory Protein/Iron Responsive Element regulatory system, which maintains cellular iron homeostasis. Activation of this system results in increased FH expression, which, in this context, appears to represent a protective response aimed at limiting iron-mediated oxidative damage. Notably, Dex markedly affects the level of PCBP1 protein, which, together with PCBP2, plays a crucial role in intracellular iron trafficking, including iron delivery to ferritin. Therefore, it can be hypothesized that, in Dex-exposed rats, disturbances in intracellular iron transport occur as a consequence of PCBP1 degradation, whereas iron transport mediated solely by PCBP2 may be insufficient. As a result, LIP may continue to increase, ultimately contributing to the elevated oxidative stress observed in our experimental model.

Dex treatment induces iron accumulation in C2C12 myotubes and promotes muscle atrophy by activating the FOXO3a-mediated ubiquitin-proteasome pathway (Jeong et al., 2025). Additionally, in skeletal muscle, Dex injection decreased AKT pathway activity, leading to FOXO transcription factor activation and the induction of Atrogin-1 and MuRF-1 (Macedo et al., 2014; Zeng et al., 2024). It is worth noting that FOXO3a, as a transcription factor, is activated by oxidative stress to protect cells from damage and promote repair mechanisms (Krafczyk and Klotz, 2022). Interestingly, inactivation of FOXO3a and MuRF-1 was observed only in EDL muscle after Dex treatment and should be considered a factor that protects against the loss of muscle proteins (Neyroud et al., 2023).

Skeletal muscle mass is regulated by multiple interconnecting pathways that control not only protein degradation but also protein synthesis (Scicchitano et al., 2018). Muscle protein synthesis is regulated by complex signaling pathways, with the mTOR pathway serving as the central regulator (Fukada and Ito, 2021). Data from this study suggest that Dex treatment partially inhibits proteins associated with muscle protein synthesis pathways. A similar observation of protein synthesis inhibition by Dex treatment was reported in rat Gastrocnemius muscle (Jhuo et al., 2023) and in myoblast cells (Cai et al., 2025).

### 4.2 The effect of acute prolonged swimming on skeletal muscle mass, oxidative stress, iron metabolism, and protein synthesis and proteolysis pathways in saline-treated rats

Acute prolonged swimming, preceded by a 4-day preparatory exercise regimen, did not influence SOL and EDL skeletal muscle and body mass, or markers of oxidative stress. These results agree with our previously published paper, in which the same swimming procedure did not induce oxidative damage in quadriceps mitochondria and, more importantly, resulted in mitoprotection, as evidenced by inhibition of mitochondrial swelling (Flis et al., 2016). Acute prolonged swimming exercise reduced PCBP1 levels, especially in the SOL muscle, suggesting inhibition of muscle regeneration of this muscle type under acute exercise conditions. Espinoza-Lewis et al. (2017) demonstrated that a hypomorphic PCBP1 mutation in mice resulted in defects in muscle growth due to impaired proliferation and differentiation of myoblasts and muscle satellite cells, and also induced the transformation of skeletal muscle myofibrils to a fast fiber type (Espinoza-Lewis et al., 2017). After acute prolonged swimming, there was also an insignificant increase in FH and FL, which may be involved in the increase in antioxidant activity of the ferritin H subunit, reduction of LIP and ROS production (Epsztejn et al., 1999; De Domenico et al., 2006), and the storage of excess labile iron (Harrison and Arosio, 1996). A similar increase in FL was observed after the same exercise protocol in rat hearts. Interestingly, significant differences were observed only in the group that underwent long-lasting swimming, but not in the group that underwent the preparatory procedure or in the group that stood for 3 hours in the water (Ziolkowski et al., 2015).

Also, specifically in the SOL muscle, acute prolonged swimming increased Atrogin-1 protein levels. An increased level of Atrogin-1 not only stimulated protein degradation but also inhibited skeletal muscle protein synthesis and regeneration (Wang et al., 2010). This is also supported by lower pmTOR in the SOL muscle, accompanied by a reduction in total p70S6K protein after acute exercise, as observed in our and others’ studies (Williamson et al., 2006). In contrast, in the EDL muscle, acute prolonged swimming did not affect AKT or pAKT but did lower MuRF-1 protein levels, thereby altering signaling pathways related to skeletal muscle protein degradation, which may protect structural proteins (Bodine and Baehr, 2014).

### 4.3 The effect of acute prolonged swimming and Dex treatment on skeletal muscle mass, oxidative stress, iron metabolism, and protein synthesis and proteolysis pathways

Dex-induced loss of muscle mass is lower after a 4-day preparatory exercise procedure, after which acute prolonged exercise was performed. A similar result was achieved in terms of compensation for the loss in muscle weight of EDL and SOL when endurance running began simultaneously with Dex treatment (Ahtikoski et al., 2004). A swimming procedure during Dex treatment did not influence oxidative stress, but did result in different tendencies in levels of proteins related to iron metabolism compared with acute prolonged swimming in saline-treated rats.

Similarly, lowering iron levels with deferoxamine prevents dexamethasone-induced muscle atrophy by reducing MuRF1 and Atrogin-1 protein levels through AKT-dependent and Krüppel-like factor 15 pathways (Jeong et al., 2025). Therefore, we can assume that restoring iron balance mitigated loss of skeletal muscle mass. Similar results were achieved when low-intensity resistance training was applied before and during Dex treatment. Resistance training attenuated the muscle atrophy effects of Dex and was associated with reductions in Atrogin-1 and MuRF-1. Interestingly, this kind of training affects only the muscles mainly recruited during it (Macedo et al., 2014), which may partially explain the different responses induced by acute prolonged swimming in SOL and EDL.

Our data show a muscle-type-specific stimulation of mTOR signaling pathways related to skeletal muscle protein synthesis in the EDL muscle. After Dex treatment, positive anti-atrophic stimulation via activation of the PI3K/Akt/mTOR signaling pathway was also observed in mice treated with small-molecule oligopeptides isolated from bovine collagen (Cai et al., 2025). Also, resistance training increases mTOR levels, thereby attenuating muscle mass loss induced by Dex (Nicastro et al., 2012; Macedo et al., 2014). Differences in response to acute swimming may be related to exercise intensity and metabolic responses after Dex treatment. In previously published data, swimming for 3 hours with the same additional load (3% of the actual body weight of the animal) generates a different intensity of exercise (different blood lactate concentration after exercise) (Flis et al., 2022), and this response may be related to impaired metabolism caused by Dex treatment (Dumas et al., 2005).

The findings of our study have to be considered in light of some limitations. Although all rats underwent 3 h of swimming with an additional load of 3% of their body weight without any assistance from laboratory staff, and we did not observe any changes in their motor function and behavior, functional measurements should be performed in future studies to verify whether Dex treatment influences not only muscle mass but also its function. Moreover, glucocorticoid administration may alter food intake and whole-body energy balance; therefore, part of the observed skeletal muscle response could potentially be secondary to changes in caloric intake.

In conclusion, our data indicate that Dex treatment reduces skeletal muscle mass, independent of muscle fiber composition. These changes are related to oxidative stress and an imbalance in iron metabolism, but differences between muscle types in the proteolysis/synthesis signaling pathway highlight contrasting responses. Acute swimming in saline-treated rats increased SOL Atrogin-1 protein levels, which, together with lower pmTOR and total p70S6K, may inhibit the signaling pathway associated with skeletal muscle protein synthesis and regeneration. In contrast, acute swimming did not affect AKT or pAKT in the EDL muscle but reduced MuRF-1 protein levels, thereby altering signaling pathways related to skeletal muscle protein degradation, which may protect structural proteins. A 4-day preparatory exercise regimen, after which acute prolonged swimming was performed, in Dex-treated rats attenuates the adverse effects of Dex treatment. Furthermore, acute prolonged swimming activates the skeletal muscle protein synthesis pathway in the EDL and inhibits MuRF-1 protein in the SOL.

## Data Availability

The original contributions presented in the study are included in the article/**Supplementary Material**. Further inquiries can be directed to the corresponding author.
