# Supplementary material for: The effect of acute prolonged swimming on skeletal muscle metabolism in different muscle types of rats after nine days of dexamethasone treatment
Source: Front Physiol. 2026 Sep 22;17:1890376. doi: 10.3389/fphys.2026.1890376 (PMC13638438; doi:10.3389/fphys.2026.1890376)
Supplement: Supplementary file 1 [file SupplementaryFile1.docx]

Supplementary Materials part 1

**Fig. S1** The effects of Dex treatment on rat Soleus (SOL) and Extensor Digitorum Longus (EDL) muscle mass.

SOL (A) and EDL (B) mass was measured in control (0) and after 3, 6, 9, and 12 days of Dex treatment. There were significant differences between the groups: ***p < 0.001, vs. 0. The data are presented as means ± SEM (n = 5 per group).

**Fig. S2** Original blot of the FH protein level and total protein image.

The marked square is the area included in the analysis.

**Fig. S3** Original blot of the FL protein level and total protein image.

The marked square is the area included in the analysis.

**Fig. S4** Original blot of the PCBP1 protein level and total protein image.

The marked square is the area included in the analysis.

**Fig. S5** Original blot of the PCBP2 protein level and total protein image.

The marked square is the area included in the analysis.

**Fig. S6** Original blot of the AKT protein level and total protein image.

The marked square is the area included in the analysis.

**Fig. S7** Original blot of the pAKT protein level and total protein image.

The marked square is the area included in the analysis.

**Fig. S8** Original blots of the FOXO3a protein level and total protein image.

The marked squares are the areas included in the analysis.

**Fig. S9** Original blot of the pFOXO3a protein level and total protein image.

The marked square is the area included in the analysis.

**Fig. S10** Original blot of the Atrogin-1 protein level and total protein image.

The marked square is the area included in the analysis.

**Fig. S11** Original blot of the MuRF-1 protein level and total protein image.

The marked square is the area included in the analysis.

**Fig. S12** Original blot of the mTOR protein level and total protein image.

The marked square is the area included in the analysis.

**Fig. S13** Original blot of the pmTOR protein level and total protein image.

The marked square is the area included in the analysis.

**Fig. S14** Original blot of the p70S6K protein level.

The marked square is the area included in the analysis.

**Fig. S15** Original blots of the pp70S6K protein level and total protein image.

The marked squares are the areas included in the analysis.
