# Supplementary material for: The effect of acute prolonged swimming on skeletal muscle metabolism in different muscle types of rats after nine days of dexamethasone treatment
Source: Front Physiol. 2026 Sep 22;17:1890376. doi: 10.3389/fphys.2026.1890376 (PMC13638438; doi:10.3389/fphys.2026.1890376)
Supplement: Supplementary file 2 [file SupplementaryFile2.docx]

Supplementary Materials part 2

**Fig. S16** Original blots of the FPN1 protein level and total protein image.

The marked squares are the areas included in the analysis.

**Fig. S17** Original blots of the APP protein level and total protein image.

The marked squares are the areas included in the analysis.

**Fig. S18** Original blots of the IGFR protein level and total protein image.

The marked squares are the areas included in the analysis.

**Fig. S19** Sample picture with the total amount of protein on the membrane.

Changes in protein levels were assessed by densitometry of immunoreactive bands and normalized to the total protein amount in the samples transferred to the membrane.

**Fig. S20** The effects of Dex treatment, swimming, and a combination of these factors on iron export proteins in rat Soleus (SOL) and Extensor Digitorum Longus (EDL) skeletal muscles.

Ferroportin 1 (FPN1) in SOL (A) and EDL (B), and Amyloid Precursor Protein (APP) in SOL (C) and EDL (D). The data are presented as means ± SEM (n = 8 in a,b, and n = 6 in c,d).

**Fig. S21** The effects of Dex treatment, swimming, and a combination of these factors on Insulin-like growth factor 1 receptor (IGF1R) in rat Soleus (SOL) and Extensor Digitorum Longus (EDL) skeletal muscles.

IGF1R was measured in SOL (A) and EDL (B) muscles. There were significant differences between the groups: ^#^p < 0.05 vs. the saline-exercised group. The data are presented as means ± SEM (n = 8 per group).
